# A Metabolomic Approach to Beer Characterization

**DOI:** 10.3390/molecules26051472

**Published:** 2021-03-08

**Authors:** Nicola Cavallini, Francesco Savorani, Rasmus Bro, Marina Cocchi

**Affiliations:** 1 Department of Applied Science and Technology, Polytechnic of Turin, Corso Duca degli Abruzzi 24, I-10129 Turin, Italy; nicola.cavallini@polito.it (N.C.); francesco.savorani@polito.it (F.S.); 2 Chemometrics and Analytical Technology, Department of Food Science, Faculty of Science, University of Copenhagen, Rolighedsvej 26, 1958 Frederiksberg C, Denmark; rb@food.ku.dk; 3 Dipartimento di Scienze Chimiche e Geologiche, Università di Modena e Reggio Emilia, Via Campi 103, 41125 Modena, Italy

**Keywords:** foodomics, beer, NMR, chemometrics, features extraction

## Abstract

The consumers’ interest towards beer consumption has been on the rise during the past decade: new approaches and ingredients get tested, expanding the traditional recipe for brewing beer. As a consequence, the field of “beeromics” has also been constantly growing, as well as the demand for quick and exhaustive analytical methods. In this study, we propose a combination of nuclear magnetic resonance (NMR) spectroscopy and chemometrics to characterize beer. ^1^H-NMR spectra were collected and then analyzed using chemometric tools. An interval-based approach was applied to extract chemical features from the spectra to build a dataset of resolved relative concentrations. One aim of this work was to compare the results obtained using the full spectrum and the resolved approach: with a reasonable amount of time needed to obtain the resolved dataset, we show that the resolved information is comparable with the full spectrum information, but interpretability is greatly improved.

## 1. Introduction

The interest expressed by the consumers towards food consumption [1] and related aspects such as production [2], food pairing [3] and consumer experience [4] has been on the rise during the last decade. As a consequence, a large number of new products is introduced into the market every year in a self-sustaining cycle of offer and demand coupled with a healthy desire to experiment. This is also true in the field of beer production where the traditional minimum recipe for brewing beer from water, malt, hops, and yeast gets constantly twisted and expanded. New approaches and ingredients are tested [5], especially by small craft breweries [6,7].

As a result, the fields of foodomics in general [8] and “beeromics” in particular [9] have been constantly growing in the recent years [10]. To meet the demand of both quick and exhaustive analytical methods for quality control [9,11] and sensory evaluation [12], many analytical techniques have been employed so far: nuclear magnetic resonance (NMR) spectroscopy [13,14,15,16,17,18,19], gas chromatography (GC) [20], gas chromatography–mass spectrometry (GC–MS) [21,22], mass spectrometry (MS) [23], near-infrared (NIR) spectroscopy [24,25,26], ultraviolet-visible (UV-Vis) spectroscopy [27,28,29], electronic tongue [30], MS electronic nose [31], middle-range infrared (mid-IR), optical-tongue [32] and fluorescence spectroscopy [33,34,35,36].

In this study, we collected and measured a set of one hundred beer samples encompassing different attributes such as origin, brewery, beer style and fermentation type; notwithstanding this variability, all beer samples were similar with respect to color (rather pale) and clearness (low turbidity). Moreover, the large majority of the samples came from industrial production. In fact, the aim of this work was to propose a methodology aiming at fast non-destructive metabolomic characterization, combining NMR spectroscopy and chemometrics, of widely consumed beer types in order to explore the compositional profile and highlight potential trends or peculiar samples, information which can be, at a successive step, matched to consumer preference. To this aim, we proposed a strategy based on multivariate curve resolution (MCR) [37] used as a resolution technique.

An MCR interval-based procedure was applied to extract chemical features from the NMR spectra [38,39,40,41], which allowed obtaining a reduced dataset of resolved relative concentrations. From the data analysis point of view, two multivariate approaches were compared: the full spectra analysis and the analysis of the chemical features extracted by MCR.

Accurate characterization of major and minor metabolites of beer consistent with previous studies is also provided in this paper (see Section 2.2.3). Evidence for the presence of trigonelline [19] confirms rather recent experimental findings which relate its origin to the addition of hops [42,43].

The results provided in this paper add to the corpus of valuable information about beer characterization that can prove to be very useful to the producers concerning both quality control and innovation.

## 2. Materials and Methods

### 2.1. Experimental

In this section, all the experimental steps for preparing the beer specimens for NMR analysis are described. First, an overview of the beer products collection is given, then the sample preparation procedure is described and finally the experimental conditions for acquiring the NMR profiles are reported.

#### 2.1.1. Sample Collection

The beer sample collection consisted of one hundred beer products that were bought from local stores. All the selected products were rather pale in color, i.e., no dark “stout-like” as well as no excessively brown beers were included in the collection. Another important criterion used for selecting the samples was the product’s clarity, in the sense that no appreciable turbidity should be seen. All the samples were different by brand, brewing style, location of production, percentage of alcohol by volume (% ABV) and color, the latter as previously described.

In Table 1, the counts for each beer style covered in the study are reported. In general, beer products can be grouped into two families based on the yeast type, namely, “top-fermented” or “ales” and “bottom-fermented” or “lagers”. These styles correspond to the yeast strains named *Saccharomyces cerevisiae* and *Saccharomyces carlsbergensis*, respectively [44].

#### 2.1.2. Sample Preparation

A collection of 2 mL vials was directly prepared from the original commercial containers (cans or glass bottles). Three small vials for each beer sample were prepared and kept frozen at −20 °C.

As the first step in sample preparation, the specimens were thawed by placing them in a water bath at room temperature. A degassing step was also performed: degassing is highly recommended by [2,45,46] as it is aimed at reducing the occurrence of measurement interferences due to bubble formation within the NMR tubes. The thawing and degassing steps were performed as follows:

- 10 min thawing in a water bath at room temperature;
- 20 min degassing in an ultrasonic bath in water at room temperature.

Since all the specimens were clear, filtration was not performed, even though this procedure is sometimes recommended in literature studies [27,47].

Preparation of the NMR tubes was executed in batches of twelve samples while keeping the unprocessed samples in a fridge at 5 °C. The newly prepared NMR tubes were placed into the instrument’s autosampler rack which, prior to spectra acquisition, was also stored in a fridge at 5 °C.

All the specimens were prepared to contain 10% D_2_O, 0.02% of sodium-3-(trimethylsilyl)propionate-d4 (TSP-d4) as a chemical shift reference [2,14,46,48] and a 20% phosphate buffer (pH = 3.55). All NMR tubes were filled with the required volume of 600 µL which was obtained by mixing 420 µL of beer specimen, 60 µL of D_2_O and 120 µL of the phosphate buffer (pH = 3.55) in H_2_O. It was reported by Duarte et al. [13] that pH values of ale and lager beers generally fall within the 3.7–4.4 interval, so the phosphate buffer was added to adjust the set of samples and obtain more homogeneous pH values, as the actual pH of the samples could not be measured. Control of pH was especially aimed at reducing the occurrence of horizontal shifts of the signals across the spectra, which may be due to the different protonation forms of compounds such as amino acids and organic acids [14,48].

The samples were prepared and analyzed by NMR following a pre-established random order.

#### 2.1.3. ^1^H-NMR Data Acquisition

All the ^1^H-NMR profiles were acquired on a Bruker Avance III 600 spectrometer (Bruker Biospin GmbH, Rheinstetten, Germany) operating at the Larmor frequency of 600.13 MHz for protons, equipped with a double-tuned cryoprobe (TCl) set for 5 mm sample tubes and a cooled autosampler (SampleJet, at 5 °C).

The spectra were acquired with TOPSPIN 2.1 (Bruker Biospin GmbH, Rheinstetten, Germany), using the NOESYGPPR1D sequence [46,48]. Presaturation of the water signal (4.77 ppm) [2,13,14,45,46,48,49,50,51] was employed, while the ethanol signals were not suppressed [14,46,48]. All the experiments were performed at 298 K with a fixed receiver gain. Each free induction decay (FID) was collected using a total of 64 scans plus four dummy scans. Acquisition time was set to 2.65 s and recycle delay was set to 6 s. Prior to Fourier transformation, the FIDs were zero-filled to 64,000 points and a 0.3 Hz Lorentzian line broadening was applied. The spectra were baseline- and phase-corrected using the TOPSPIN built-in processing tools. This correction was performed automatically for all spectra and then, depending on the obtained results (assessed by a trained NMR user), a further manual adjustment was performed when strictly necessary. For all spectra, the ppm scale was referenced to the TSP peak (0.00 ppm). The spectral window was set to 20.5 ppm.

### 2.2. Data Preprocessing and Data Analysis Methods

This section describes all the data analysis steps from raw spectra preparation to multivariate analysis. The preliminary preprocessing of NMR spectra described in Section 2.2.1 is common to the analyses of both the full spectra and resolved features datasets. In Section 2.2.2, Section 2.2.3 and Section 2.2.4, the specific procedures applied for features resolution are described. Finally, in Section 2.2.5, the multivariate data analysis and preprocessing methods used in the study are reported.

#### 2.2.1. ^1^H-NMR Data Preparation

The raw NMR spectra were imported and processed under the MATLAB environment. The spectra were first globally denoised (smoothed) using a simple moving average algorithm [52] (window width = 3, polynomial order = 0): this step was performed in the perspective of working by focusing on small portions of the whole spectral width using the so-called “interval-based” approach [53].

Then, a set of manually chosen small intervals was defined, each interval containing single peaks or small groups of peaks, to allow better signal resolution (as explained in Section 2.2.2). Finally, each interval was aligned using the icoshift tool [54,55]. The aligned intervals were merged and used for the analysis of the full spectra, but they also constitute the basis for the peak resolution by MCR, as described in Section 2.2.2.

#### 2.2.2. ^1^H-NMR Spectra Peaks’ Resolution by MCR

Since NMR spectra carry different information in different spectral regions, it is common to roughly split them into three regions [13,53]: aliphatic/organic acids (0–3 ppm), carbohydrates (3–5 ppm) and aromatic (6–9 ppm) regions. These regions mainly differ because of baseline noise, the signals’ average intensities and the involved molecules [53]. An interval-based approach allows effectively handling those differences, leading to meaningful chemical quantification of the metabolites, also taking advantage of improved interpretability and model performance.

In the framework of an interval-based approach, instead of building one overall model based on the whole spectral width, a set of 53 interval-specific MCR models was built. In order to choose the correct number of components, four MCR models for each interval were built, using from two to five components, by means of an in-house written routine. A list of all the integrated intervals with their boundaries (in ppm), model complexity and selected components is provided in the Supplementary Materials (Table S1). Regarding the settings for MCR modelling, the maximum number of iterations was set to 1000 and the non-negativity constraint was applied both in the rows and columns directions.

**Figure 1:**
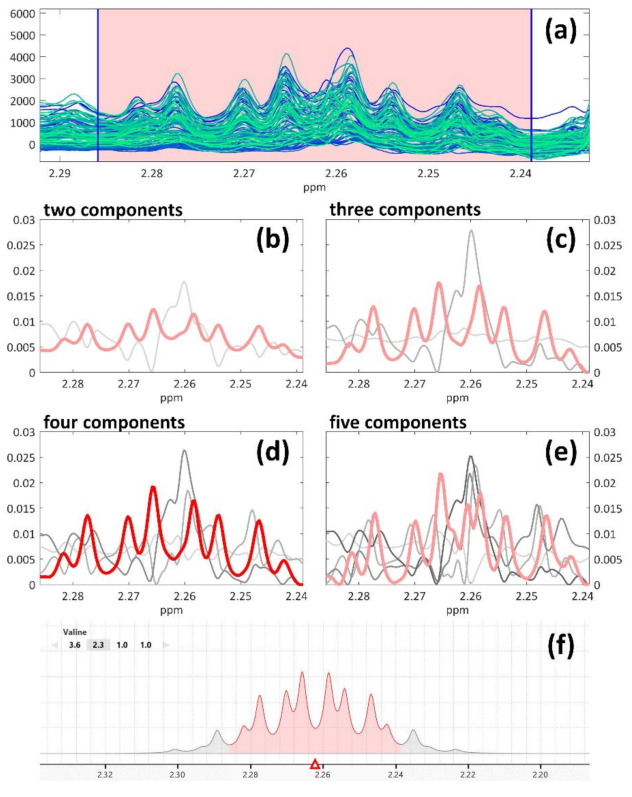
The identification procedure: an example from the amino acid valine. The aligned raw data shown in (**a**) constitute the input for MCR modelling. Then, four models were built using from two to five components (**b**–**e**). Based on literature sources, the initial hypothesis for the signal’s assignment was “valine” [14] whose spectral profile provided by Chenomx, represented here as a screenshot from the Profiler window of the software (**f**), could be matched in each model with the resolved profile highlighted in red.

Each set of resolved profiles corresponding to the four MCR models was plotted as shown in Figure 1b–e, allowing for clear comparisons between the models: such a visual representation allowed the identification of the best model and the selection of the resolved components related to the chemical information. All the other components describing background effects, noise or signals not related to NMR peaks were excluded. The integrated area provided by MCR was carefully evaluated for each selected component before generating the final features dataset (Section 2.2.4).

An example of the MCR peak resolution and identification process is shown in Figure 1, investigating the amino acid valine which is characterized by a quite complex spin system resulting in a symmetric multiplet. Its presence in beer was reported by many bibliographic sources [13,20,43,46,50,56], even though only Nord et al. also reported its chemical shift and assignment [14]. In the example, four MCR models are shown: in each of them, it is possible to identify one resolved spectral profile that matches the actual complex signal of valine (in red in Figure 1b–e), whose correct profile was recovered from the reference library of Chenomx (Figure 1f, a screenshot from the software’s interface). It is interesting to notice how different numbers of components affect the extraction performance of the compound’s profile. For instance, the signal related to valine is already recognizable in the first model (fitted with two components, Figure 1b), even though a vertical offset is also present: in this case, the piece of information related to the compound of interest may need further “cleaning”, i.e., noise or background effects should be taken care of. In the inspected models built with three and four components (Figure 1c–d), which basically show identical performance, the vertical offset disappeared. The last model was fitted with five components (Figure 1e); also, in this case, the correct spectral profile of valine was recovered, but some artefacts appeared at the center of the multiplet. Moreover, the signal itself lost its nice symmetry that was recovered by the previous models, thus indicating that five components do not correspond to the correct model dimension. Based on these considerations, the three-component model was selected, and the resolved profile was identified as valine.

#### 2.2.3. ^1^H-NMR Peak Identification and Assignment

**Figure 2:**
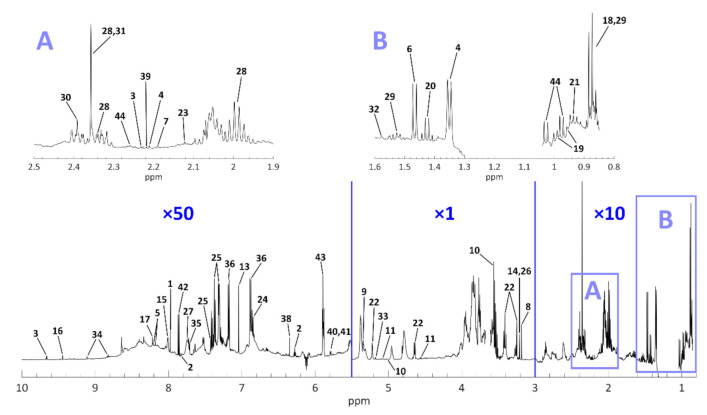
The average spectrum of the ales of the dataset, with all the identified signals (full assignments are reported in Table 2). To improve visual clarity, the signals of the aromatic zone (5.5–10 ppm) and the aliphatic zone (0.8–3 ppm) were multiplied respectively by a factor of 50 and 10. Moreover, expansions of the regions labelled “**A**” and “**B**” are provided in the upper part of the figure.

As anticipated in Section 2.2.1, tentative identification of the extracted compounds was performed by comparing the resolved NMR signals to literature sources [2,13,14,17,19,20,43,45,46,48,50,53,56,57,58]. To make some identifications and assignments more robust, the information provided by the reference library of Chenomx and the NMR spectra searchable on the Human Metabolome Database (HMDB, [59]) were also used. The level of confidence in metabolite assignment can be assessed using the framework defined by the Chemical Analysis Working Group of the Metabolomics Standards Initiative [60,61], in which our work would approximately correspond to level 2 (i.e., “Putatively annotated compounds”). All assignments are reported in Table 2, whose last column contains the literature sources that justify each identification. All the entries of Table 2 are also depicted in Figure 2, which shows the positions of all resolved signals on the averaged spectrum obtained from the whole beer dataset. For more precise visual representation of all the resonance signals which were processed with MCR, see Figure S1 in the Supplementary Materials.

#### 2.2.4. Constitution of the Features Dataset

The result of the resolution process consisted of 63 resolved components whose relative concentrations obtained by MCR were merged to generate a new dataset, hereinafter simply referred to as the “features dataset”. Fifty-nine of these features were tentatively assigned as explained in Section 2.2.3. The remaining four unidentified features were labelled “unknown”, as reported in Table 2.

#### 2.2.5. Multivariate Data Analysis Methods and Dataset Preprocessing

Principal component analysis (PCA) [62,63] was used for exploratory purposes, on both the full spectrum and features datasets. As explained in the previous sections, multivariate curve resolution (MCR) [64,65] was used for extracting the chemical features. Pareto scaling was used to preprocess the NMR spectra dataset [66], while autoscaling was used to preprocess the features dataset (i.e., integrated areas of resolved components).

### 2.3. Software

The whole data analysis process was carried out on MATLAB 2017b (Mathworks, Natick, MA, USA). NMR interval resolution by MCR, PCA exploratory analysis and Pareto scaling preprocessing were performed using the respective functions contained in PLS_Toolbox (version 8.6, Eigenvector Research Inc., Manson, WA, USA). NMR spectral alignment was operated using icoshift [54,55] (http://www.models.life.ku.dk/icoshift, date of last access: 17 December 2020). In-house written routines were used to jointly process the icoshift alignment and MCR steps. Identification of the resolved components was in part based on the digital library of Chenomx using the Profiler GUI included in the Chenomx NMR Suite (version 8.3, Chenomx Inc., Edmonton, Alberta, Canada, https://www.chenomx.com, date of last access: 17 December 2020).

## 3. Results and Discussion

**Figure 3:**
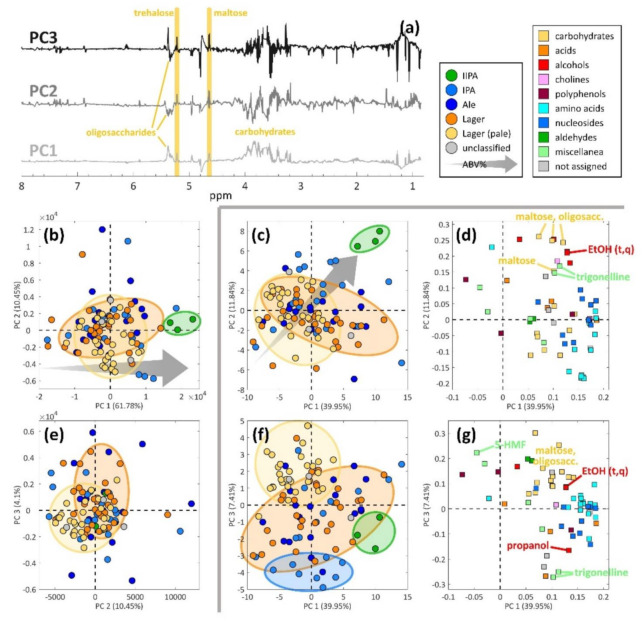
Comparison of the spectral (**a**,**b**,**e**) and features (**c**,**d**,**f**,**g**) PCA results. The score plots (**b**,**c**,**e**,**f**) are colored according to the beer style, while the metabolites reported in the loadings plots of the features dataset (**d**,**g**) are colored according to their chemical class. The grey arrow reports the direction of the ABV content trend, from lower to higher.

The discussion of the results is focused on the groupings related to beer styles and the trend linked to the alcohol content (% ABV). The most meaningful principal components (PCs) are discussed and reported in Figure 3. The solid grey line in the figure separates the full spectrum from the features dataset results. In addition to PCA, in order to get a deeper insight of the similarity and clustering tendency of the studied beer samples, other unsupervised methods such as projection pursuit (PP), t-distributed stochastic neighboring entities (t-SNE) and co-clustering were applied obtaining results inferior (PP) or similar to PCA (t-SNE, co-clustering). Analysis based on co-clustering [67] (via penalized matrix decomposition [68]), while efficient to visualize in a parsimonious way the main groups, did not yield extremely clear results. For the sake of clarity and to keep the discussion of this section as smooth as possible, the co-clustering results were included and briefly discussed in the Supplementary Materials (Figure S2).

Starting from the information about the beer styles, they appear completely overlapped when inspecting the PCA model of the full spectrum dataset, as can be seen in the score plots of Figure 3b,e. The first three and most important components (which capture 76.33% of the dataset’s variance) are therefore not able to provide a clear grouping trend related to the beer style. On the contrary, in the case of the features dataset, the beer style information appears rather overlapped with PC1 vs. PC2 (Figure 3c), but becomes clearer by inspecting PC1 vs. PC3 (Figure 3f). PC3 of the features dataset model is able to separate the pale lagers (in yellow) from the lagers (in orange) and also a group of IPAs (in light blue), which is recognizable in the lower part of the plot, at negative values on PC3. However, the lager samples (in orange) appear very overlapped with the majority of the ale samples (in blue) in all the plots reported in Figure 3.

The metabolites that are responsible for grouping the pale lagers (in yellow in Figure 3f) are shown in Figure 3g, in which the PC1 vs. PC3 loadings of the features dataset are reported. Beers from producers such as Hite, Oriental Brewery, Heineken, Budwiser, Pilsner Urquell and San Miguel are present in this group: these are very widespread products, and their style generally does not involve much addition of hops or spices. These pale lagers are mainly characterized by compounds related to sugars, such as oligosaccharides and trehalose, and malt, which is the main source of polyphenols, as 70–80% of their total amount in beer comes from malt [69]. Another important metabolite turns out to be acetaldehyde, a well-known beer flavor [20]. In addition to these compounds and coherently with their style, the pale lager samples are characterized by a number of metabolites located in the opposite direction in the loadings plot, at negative PC3 scores (Figure 3g): compounds such as propanol and trigonelline, a compound derived from hops [43], are found to be present in very low amounts.

Another interesting metabolite that proves important for the pale lager samples is 5-hydroxymethylfurfural (5-HMF). This compound is a known marker of beer aging [46] and since these lager samples seem to have a higher content of it, they might be more prone to fast aging than other beer styles, in the sense that their organoleptic characteristics may already be more deteriorated than of other beers whose content of 5-HMF is found lower.

Remarkably, in the lower part of the plot of Figure 3f, a clear group of IPAs is found, together with two ales. No correspondence with a similar grouping in the full spectrum case could be found. These products mainly come from breweries like Mikkeller and To Øl that tend to experiment a lot, especially using different varieties and combinations of hops.

As previously discussed, trigonelline (lower part of Figure 3g) is among the most influential metabolites for distinguishing the pale lager group and the IPA group. Trigonelline is very interesting [19,43,56] and it has been recently identified in beer and described as a plant-associated metabolite whose concentration increases with boiling [43]. Hops are generally added right before boiling the beer wort, so that the alpha acids can be extracted from the raw hops and thermally isomerized into iso-alpha acids, giving the beer its characteristic bitter taste [70]. For these reasons, trigonelline is a metabolite that can be associated with hops.

Two unknown metabolites are found very close to trigonelline (Figure 3g), and further research may be needed to assess whether they are “rare compounds” which may also arise from hops or added spices, given their position in the loadings plot.

A clear small group of three IIPAs can be identified in both the full spectrum and features datasets, as highlighted with green circles in Figure 3b,c,f. In the case of the features dataset, the IIPA group can be more clearly seen by inspecting the PC1 vs. PC2 score plot (Figure 3c), while in the PC1 vs. PC3 plot, the three samples are still close to each other, but their position is not very distant from the rest of the samples: they look, therefore, more similar to the bulk of the samples. The most influential metabolites for this group, as inferred from the PC1 vs. PC2 loadings plot in Figure 3d, resulted to be ethanol, some higher alcohols (isopentanol, isobutanol, propanol), malt-related compounds (maltose, oligosaccharides) and trigonelline. Given the beer style, which is characterized by higher ethanol content and stronger taste, high contents of these compounds can be expected, as they are related to beers with strong taste and a wider flavor bouquet.

Regarding the alcohol content, a trend related to the ABV content can be identified in both datasets, as the grey arrows show in Figure 3b,c. It is important to notice that the numbers of light, medium and strong beers are rather unbalanced, with the medium (3.5–6% ABV) beers representing the large majority of the samples.

In the case of the full spectrum dataset, PC1 seems more directly related to the ABV content (grey arrow in Figure 3b), with the light beers located at negative scores and the strong ones located at positive scores. The most influential spectral variables on PC1 are mainly related to the carbohydrates region (3–5.5 ppm) and, more specifically, to the signals of maltose, trehalose and oligosaccharides (Figure 3a). However, the same signals also have some importance related to the other two PCs, but the loadings’ directions appear less clear.

By inspecting the results from the features dataset, it can be noticed that both PC1 and PC2 describe the ABV trend (Figure 3c): the lighter samples are generally located at negative values on both components while the strong samples are located at positive scores on both components (Figure 3c). The metabolites mainly responsible for this trend are reported in the loadings plot in Figure 3d: in general, it appears that the stronger the beer, the higher the overall content of all metabolites, as a sort of “leading effect” related to ABV. This could be explained considering that fermentations which yield more ethanol generally last longer and also generate more and larger varieties of compounds, i.e., a richer flavor bouquet is obtained. As expected, the most influential compounds correspond to alcohols (both ethanol and higher alcohols) and malt-related metabolites along with trigonelline, as previously discussed when commenting on the IIPA samples.

It is worth noting that at least six carbohydrate-related metabolites are found at negative scores on PC2 (Figure 3d). This may be due to the fact that malt is a fundamental ingredient in the recipe for brewing beer, as its characteristics are responsible for many flavor aspects of the final product. For instance, ale beers (including IPAs and IIPAs) are usually brewed with darker malts, whose production involves a more intense roasting treatment that generates more intense colors and stronger taste. The lagers, on the contrary, are generally brewed with lighter malts, which yield a more bready taste and flavor to the product. More precise peak assignments of the carbohydrate-related variables, which are beyond the scope of the present paper and would probably need much more information (e.g., planning a study based on 2D NMR spectra), may shine a light on potential differences regarding the type of malt and its characteristic metabolites.

## 4. Conclusions

We have illustrated the potential and efficiency of using an interval-based approach, especially from the point of view of the grouping information that can be extracted from a set of NMR spectra, when properly processed. The peak-by-peak processing procedure allows considering all the interval-specific signals systematically. Since MCR models are built automatically, the user’s intervention is limited to defining the intervals and then choosing between a small set of models: this can be very practical to make use of the analyst’s expertise in spectroscopy and chemistry, as well as perform a sort of “internal validation” of the actual content of chemical information while processing the data. Moreover, it is worth noting that the approach applied in this study generally provides at least the same information as the traditional approach of peak identification without the resolution step, but the processed information is made simpler and therefore clearer and more easily interpretable.

This type of approach, in which chemometrics is coupled with NMR spectroscopy, provided clear insights into the composition of beer and helped shed some light on the rich and complex NMR data. As a result, a rather detailed metabolomic characterization of the set of beer samples was obtained and easily interpreted. The obtained information, together with previous studies, can be used as a basis for a better understanding of beer composition, especially of how the main differences and global effects due to the macroscopic characteristics of beer reflect on its characteristics at the microscopic level (i.e., relative to chemical compounds and metabolites). This approach can therefore be useful, e.g., for producers, who may use the gathered information for further recipe optimization aimed at meeting the consumers’ demand for interesting and innovative products. This could be implemented in the form of preference mapping, e.g., using online consumer evaluations.

Finally, from the point of view of signal identification and assignment, further developments should focus on obtaining and validating more specific and detailed chemical features. For instance, information about J-coupling values or the anomerization of oligosaccharides could be investigated by means of dedicated experimental plans and NMR experiments (encompassing 2D and 1D TOCSY NMR acquisitions also using standard reference compounds). This also holds true with the most important metabolites that were highlighted by our analysis.

## Figures and Tables

**Table 1:** Number of samples for each fermentation style (i.e., yeast strain) and for each beer style.

Fermentation Style (Yeast Strain)		% ABV Range	Beer Styles	
Top, “ales” *(S. cerevisiae)*	40	5.7 ± 1.3%	Ale	18
			India pale ale (IPA)	19
			Imperial India pale ale (IIPA)	3
Bottom, “lagers” *(S. carlsbergensis)*	57	4.8 ± 1.0%	Lager	30
			Lager (pale)	27
Unclassified ^1^	3	4.8–5.2–6.0%	Organic ginger brew–Oktoberfest–Kristallweizen	

^1^ These beer products are brewed with either *Saccharomyces cerevisiae* or *carlsbergensis* yeast, but their style is generally different from the two large “top” and “bottom”-style families.

**Table 2:** List of resolved compounds with tentative names, chemical shifts (δ, ppm), signal multiplicity and assignment and references supporting the signal’s identification.

	Tentative Compound Name	Chemical Shift (δ, ppm)	Multiplicity and Assignment	References ^c^
1	2′-Deoxyguanosine ^b^	7.98	s	[56] ^b^; Chenomx; HMDB0000085
2	2′-Deoxyuridine ^a^	7.83	d	[56] ^b^; Chenomx; HMDB0000012
		6.28	t	
3	Acetaldehyde ^a^	9.66	q, CHO	[13]; HMDB0000990
		2.23	d, CH3	
4	Acetoin ^b^	2.21	s, COCH3	[20,43,58] ^b^; Chenomx; HMDB0003243
		1.35	d, CH3	
5	Adenine	8.21	s	[57] (not 8.21 but 8.32 ppm); Chenomx
		8.18	s	
6	Alanine	1.47	d, β-CH3	[13,14,17,19]; Chenomx
7	Butanone ^a^	2.19	s	Chenomx
8	Choline	3.18	s, N-CH3	[57]; Chenomx
9	Oligosaccharides I	5.35	C1H glyc. bond	also called “dextrins” in [13]
10	Oligosaccharides II ^a^	3.54		also called “dextrins” in [17]
		5.01	d	
11	Oligosaccharides III ^a^	5.08	d	also called “dextrins” in [45] or generally “carbohydrates” in [14,53]
		5.08	d	
		4.57	m	
12	Ethanol	3.64	q, CH2	[2,13,14,17,45,46,50]
		1.17	t, CH3	
13	Gallic acid	7.04	s, C2H, C6H	[13,19,46]; Chenomx
14	Glucose ^a^	3.22	dd	Chenomx; HMDB0000122
15	Guanosine ^b^	8.00	s	[56] ^b^, [19]; Chenomx; HMDB0000133
16	5-Hydroxymethylfurfural ^a^	9.44	s	[46]; HMDB0034355
17	Inosine	8.22	s, C4′H	[13,19]; Chenomx
18	Isobutanol/Isopentanol	0.88	d, CH3	[13,17]
19	Isoleucine	0.99	d, ε-CH3	[14]; Chenomx
		0.95	t, δ-CH3	
20	Isopentanol	1.42	CH	[13,48]
21	Leucine	0.96	t, δ-CH3	[14]; Chenomx; HMDB0000687
22	Maltose	5.22	d, α-C1H	[13]; Chenomx
		4.64	d, β-C1H	
		3.42	dd, α/β-C4H	
		3.26	dd, β-C2H	
23	Methionine ^a^	2.12	m, β-CH2	[14]; Chenomx
24	N-acetyltyrosine ^a^	6.84	m	Chenomx; HMDB0000866
25	Phenylalanine	7.43	m, H2/H6	[14,19,50]; Chenomx
		7.37	m, H4	
		7.33	m, H3/H5	
26	Phosphocholine	3.22	s, N-CH3	[57]; Chenomx
27	Polyphenols ^a^	7.74		[2]
		7.75		
		7.77		
28	Proline	2.36	m, β-CH2	[13,14,17,48,57]; Chenomx
		2.34	m, β-CH2	
		1.98	m, γ-CH2	
29	Propanol	1.53	m, CH2	[13]; HMDB0000820
		0.88	t, CH3	[13,46]
30	Pyroglutamate ^a^	2.39	m, CH2	[43,56] ^b^; Chenomx; HMDB0000267
31	Pyruvate	2.36	s, CH3	[2,13,14,17,46,48]; Chenomx
32	Pyruvate hydrate ^a^	1.58	s, CH3	[14]
33	Trehalose	5.18	d	[2]; Chenomx
34	Trigonelline ^b^	9.11	s	[19,43,56] ^b^; Chenomx
		8.82	m	
35	Tryptophan	7.72	bd, C4H	[13,14,19,50]; Chenomx
36	Tyrosine	7.18	d, C2H, C6H	[13,14,17,19,50,57]; Chenomx
		6.89	d, C3H, C5H	+ [2]
37	Unknown 1	10.2	s	
38	Unknown 2 ^d^	6.35	s	[46] ^d^
39	Unknown 3	2.22	s	
40	Unknown 4	5.79	m	
41	Uracil^b^	5.79	d	[56] ^b^, [19]; Chenomx; HMDB0000300
42	Uridine	7.86	d, C6H	[2,13,17,19,50]; Chenomx
43	Uridine/Guanosine ^b^	5.89	m, C1′H	[13,17,50,57], [56] ^b^; Chenomx; HMDB0000296 (Uri), HMDB0000133 (Guan)
44	Valine	2.26	m, β-CH	[14,19]; Chenomx
		1.03	d, γ-CH3	
		0.97	d, γ-CH3	

^a^ uncertain identification; ^b^ reported without information about chemical shift and multiplicity; ^c^ confirmed by inspecting the ^1^H-NMR spectrum from the HMDB; ^d^ signal found but not identified in [46].

## Data Availability

The data presented and analyzed in this study are available in the Supplementary Materials in the MATLAB format (.mat).

## Supplementary Materials

The following items are available online, Figure S1: Expansions of the spectral zones containing the identified signals (as reported in Table 2); Figure S2: Co-clustering results obtained from the features dataset; Table S1: List of resolved intervals and related selected components.

## Sample Availability

Samples are not available.
