# Supplementary material for: A Metabolomic Approach to Beer Characterization
Source: Molecules. 2021 Mar 8;26(5):1472. doi: 10.3390/molecules26051472 (PMC7962951; doi:10.3390/molecules26051472)
Supplement: Supplementary file 1 [file molecules-26-01472-s001.pdf]

molecules MDPI

Supplementary Materials

A Metabolomic Approach to Beer Characterization

Nicola Cavallini[1], Francesco Savorani[1], Rasmus Bro[2] and Marina Cocchi[3,*]

[1] Department of Applied Science and Technology, Polytechnic of Turin, Corso Duca degli Abruzzi 24, I-10129, Turin, Italy; nicola.cavallini@polito.it, francesco.savorani@polito.it
[2] Chemometrics and Analytical Technology, Department of Food Science, Faculty of Science, University of Copenhagen, Rolighedsvej 26, 1958, Frederiksberg C, Denmark; rb@food.ku.dk
[3] Dipartimento di Scienze Chimiche e Geologiche, Università di Modena e Reggio Emilia, Via Campi 103, 41125, Modena, MO, Italy; marina.cocchi@unimore.it
* Correspondence: marina.cocchi@unimore.it

Table of Contents

Raw data file. Description of the content and organization of the raw data made available with the paper.

Table S1. List of resolved intervals and related selected components.

Figure S1. Expansions of the spectral zones containing the identified signals (as reported in Table 2).

Figure S2. Co-clustering results obtained from the features dataset.

Raw data file

The raw data are organized in a MATLAB file (molecules-1080756_raw_data.mat) as follows:

- beer_NMR_aligned_spectra (100 × 29936, dataset object) – the aligned spectra;
- beer_NMR_features (100 × 63, dataset object) – the 63 features extracted using the MCR procedure detailed in Section 2.2.2;
- ABV (100 × 1, double) – a numerical vector containing the alcohol-by-volume % values corresponding to each sample.

The two datasets share the same sample order, sample label sets ("coded name", "product name"), class information ("top–bottom", "ale–lager", "brewery", "beer style"). The class information about the beer style corresponds to the groups represented in Figures 3 and S2.

The features dataset also contains two more sets of class information related to the variables (i.e., the extracted features):

- chemical class is organized according to the main chemical function of the identified compound;
- general resonance zone is defined according to the NMR spectral window partition into three zones—aliphatics, carbohydrates, aromatics—as detailed in Section 2.2.2 and in [13,53].

The dataset object is freely available and can be downloaded from:

https://www.mathworks.com/matlabcentral/fileexchange/39336-dataset-object

Table S1. List of resolved intervals and related selected components. Numbers in the "Selected components" column should be read as "first component", "second component" and so on, as they are referred to the actual MCR-resolved components which were identified as meaningful chemical signals in each individual model.

Interval number	Interval start (ppm)	Interval end (ppm)	Model complexity (number of components)	Selected components
1	10.16	10.24	2	1

2	9.65	9.68	2	2
3	9.44	9.45	3	2
4	9.10	9.13	3	2
5	8.79	8.86	2	2
6	8.20	8.23	3	1, 3
7	8.17	8.19	3	2
8	7.99	8.02	2	1
9	7.97	7.99	2	2
10	7.85	7.88	2	1
11	7.82	7.85	2	2
12	7.70	7.79	4	1–4
13	7.29	7.45	2	1, 2
14	7.16	7.20	2	1
15	7.04	7.05	2	2
16	6.80	6.91	2	1, 2
17	6.34	6.36	2	1
18	6.25	6.31	2	2
19	5.87	5.91	2	2
20	5.77	5.80	3	2, 3
21	5.35	5.42	3	1, 2, 3
22	5.20	5.23	2	2
23	5.16	5.19	2	1
24	5.06	5.10	3	1, 3
25	5.00	5.03	2	1
26	4.60	4.69	2	2
27	4.54	4.59	3	1
28	3.50	3.60	2	1, 2
29	3.37	3.45	3	1
30	3.24	3.28	2	1
31	3.20	3.24	2	1, 2
32	3.18	3.19	2	1, 2
33	2.37	2.42	2	1, 2
34	2.35	2.37	2	1, 2
35	2.29	2.35	2	1, 2
36	2.24	2.29	3	2
37	2.22	2.24	2	2
38	2.20	2.22	4	3, 4
39	2.18	2.20	2	2
40	2.12	2.13	2	2
41	1.94	2.00	2	1, 2
42	1.56	1.59	3	2
43	1.49	1.56	2	2
44	1.45	1.48	2	1
45	1.40	1.45	2	2
46	1.32	1.38	2	2
47	1.02	1.04	2	2
48	0.98	1.01	2	2
49	0.96	0.99	2	1
50	0.91	0.96	2	2
51	0.85	0.90	2	1, 2
52	3.62	3.67	1	1

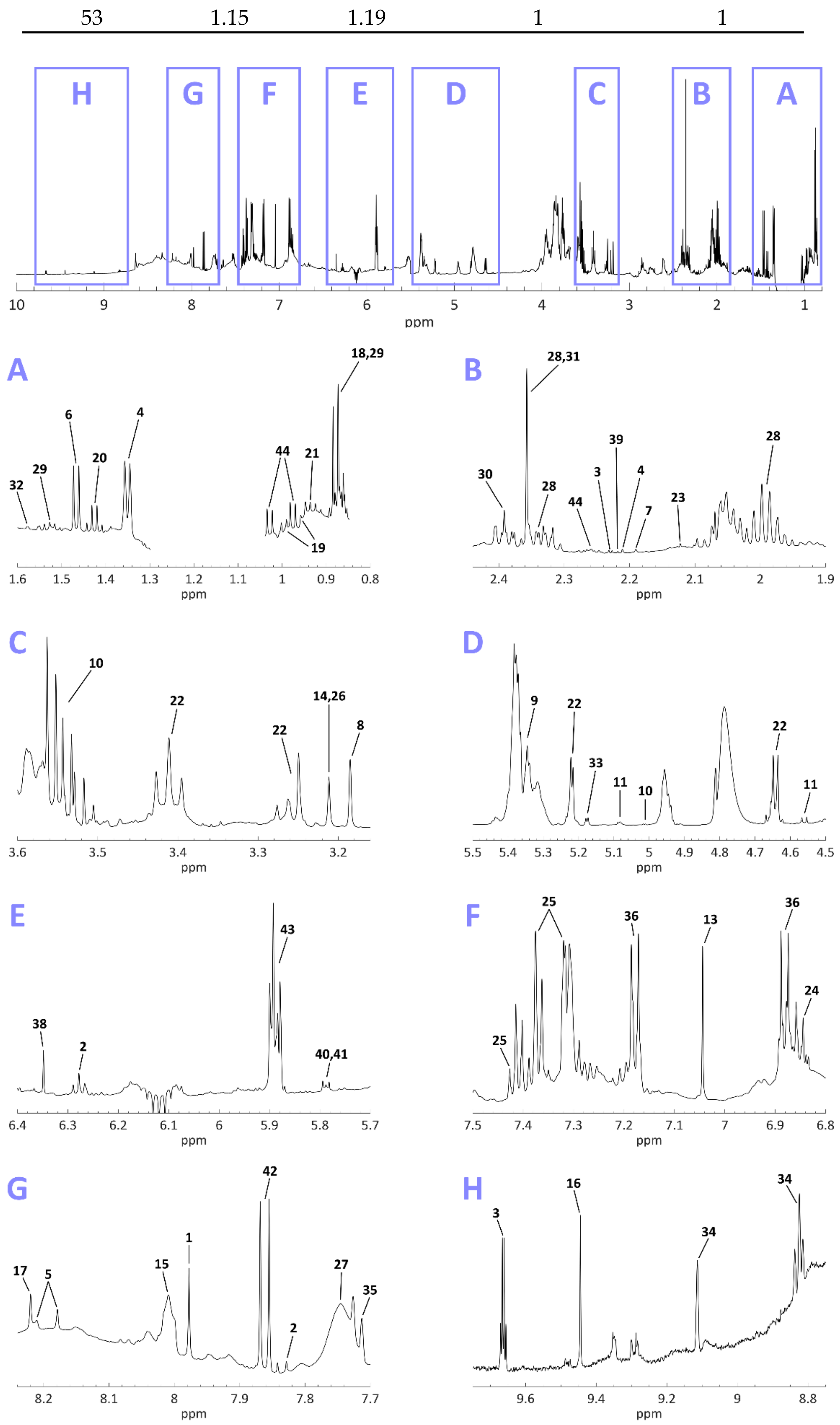
53
1.15
1.19
1
1
H
G
F
E
D
C
B
A
10
9
8
7
6
5
4
3
2
1
ppm
A
18,29
6
4
29
20
32
44
21
19
1.6
1.5
1.4
1.3
1.2
1.1
1
0.9
0.8
ppm
B
28,31
39
28
30
28
3
4
44
7
23
2.4
2.3
2.2
2.1
2
1.9
ppm
C
10
22
14,26
22
8
3.6
3.5
3.4
3.3
3.2
ppm
D
9
22
22
33
11
10
11
5.5
5.4
5.3
5.2
5.1
5
4.9
4.8
4.7
4.6
4.5
ppm
E
43
38
2
40,41
6.4
6.3
6.2
6.1
6
5.9
5.8
5.7
ppm
F
25
36
13
36
24
25
7.5
7.4
7.3
7.2
7.1
7
6.9
6.8
ppm
G
42
1
17
5
15
27
35
2
8.2
8.1
8
7.9
7.8
7.7
ppm
H
16
34
3
34
9.6
9.4
9.2
9
8.8
ppm

Figure S1. This Figure is complementary to Figure 2: expansions of the spectral zones containing the identified signals (as reported in Table 2) are depicted to make it clearer where those signals are and how they look. To do so, the average spectrum of the ales of the dataset was plotted and it is represented in its entirety in the top part of the Figure.

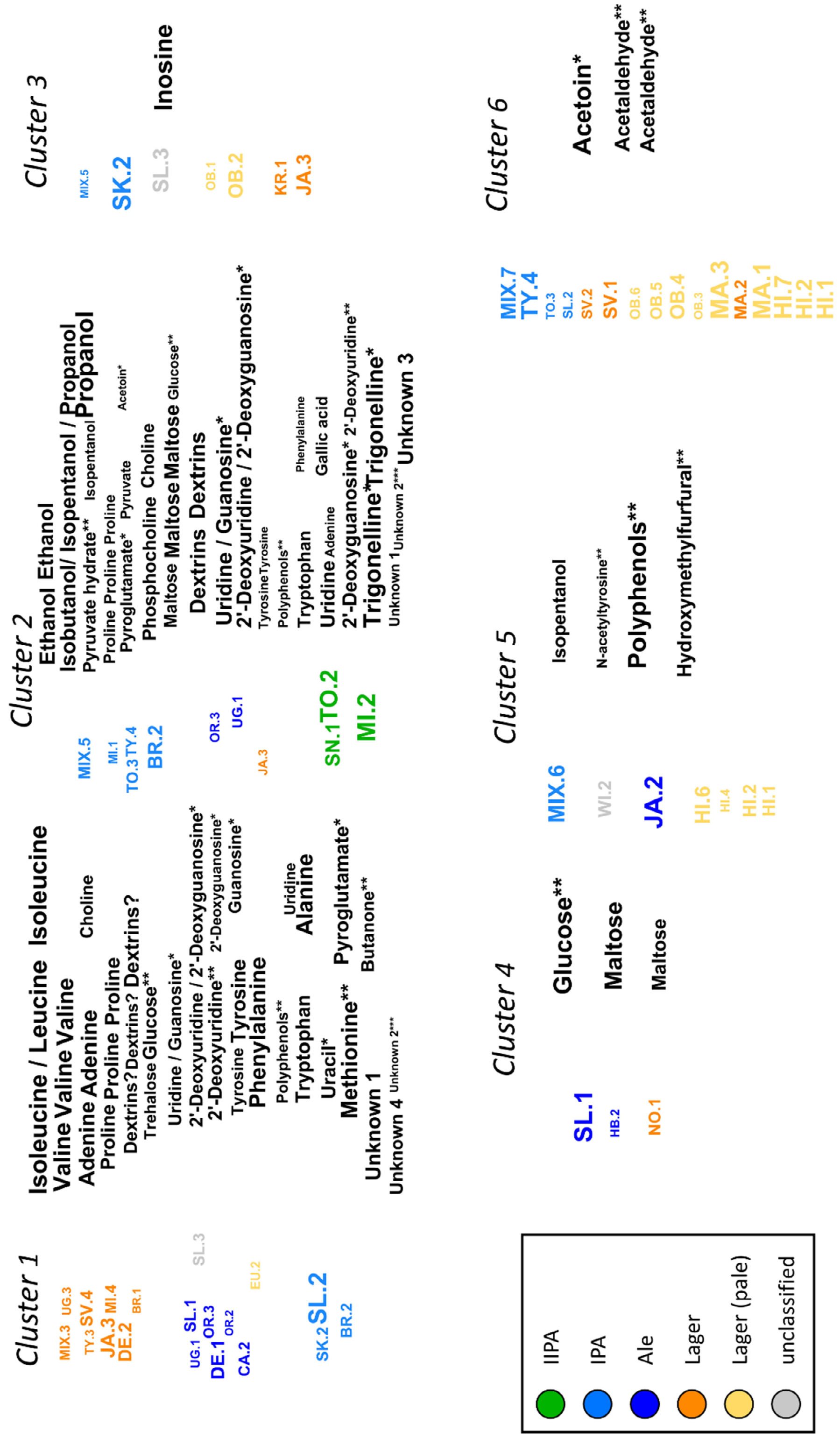

Figure S2. Co-clustering [67] results. The model was fitted with six co-clusters on the autoscaled features dataset. For each cluster, a set of samples and a set of relevant variables are defined. The assignment to a cluster is not exclusive, i.e., any sample or variable may appear in multiple clusters. Text size is related to the influence of each sample and variable on their cluster.

Figure 2S: Brief Discussion of the Results

Cluster 1: seems to correspond to the samples in Figure 1c at positive PC1 scores and negative PC2 scores, in a direction strongly determined by amino acids and some carbohydrates.

Cluster 2: corresponds to beers with generally high alcohol content, therefore, many of these samples can be found in Figure 3c at positive PC1 and PC2 scores (the area to which the ABV arrow is pointing to). It is interesting to notice that a large number of compounds are found relevant and, above all, metabolites such as alcohols (ethanol, isobutanol, isopentanol and propanol), carbohydrates (maltose, glucose and "dextrines", i.e., oligosaccharides) and trigonelline appear to be the most influential, probably as a result of the inclusion of the three IIPA beers in the cluster.

Cluster 6: corresponds to the trend highlighted in Figure 3f, where the lager (pale) class is found at positive PC3 scores.
